# Effects of androgen deprivation on brain function in prostate cancer patients – a prospective observational cohort analysis

**DOI:** 10.1186/1471-2407-12-371

**Published:** 2012-08-27

**Authors:** Herta H Chao, Edward Uchio, Sheng Zhang, Sien Hu, Sarah R Bednarski, Xi Luo, Michal Rose, John Concato, Chiang-shan R Li

**Affiliations:** 1 Department of Internal Medicine & Yale Comprehensive Cancer Center, Yale University School of Medicine, New Haven, CT 06519, USA; 2 Medical Service, VA Connecticut Health Care System, West Haven, CT 06516, USA; 3 Tower Research, Los Angeles, CA, 90048, USA; 4 Department of Psychiatry, Yale University School of Medicine, New Haven, CT, 06519, USA; 5 Department of Biostatistics, Brown University School of Medicine, Providence, RI, 02912, USA; 6 Clinical Epidemiology Research Center, West Haven, CT, 06516, USA; 7 Department of Neurobiology, Yale University School of Medicine, New Haven, CT, 06520, USA; 8 Interdepartment Neuroscience Program, Yale University, New Haven, CT, 06520, USA; 9 Cancer Center, VA Connecticut Health Care System, 950 Campbell Avenue, West Haven, CT, 06516, USA

**Keywords:** Androgen deprivation, Prostate cancer, Brain function, Cognitive function

## Abstract

**Background:**

Despite a lack of consensus regarding effectiveness, androgen deprivation therapy (ADT) is a common treatment for non-metastatic, low-risk prostate cancer. To examine a particular clinical concern regarding the possible impact of ADT on cognition, the current study combined neuropsychological testing with functional magnetic resonance imaging (fMRI) to assess both brain activation during cognitive performance as well as the integrity of brain connectivity.

**Methods:**

In a prospective observational cohort analysis of men with non-metastatic prostate cancer at a Veterans Affairs medical center, patients receiving ADT were compared with patients not receiving ADT at baseline and at 6 months. Assessments included fMRI, the N-back task (for working memory), the stop-signal task (for cognitive control), and a quality of life questionnaire.

**Results:**

Among 36 patients enrolled (18 in each group), 30 completed study evaluations (15 in each group); 5 withdrew participation and 1 died. Results for the N-back task, stop-signal task, and quality of life were similar at 6 months vs. baseline in each group. In contrast, statistically significant associations were found between ADT use (vs. non use) and decreased medial prefrontal cortical activation during cognitive control, as well as decreased connectivity between the medial prefrontal cortex and other regions involved with cognitive control.

**Conclusions:**

Although ADT for 6 months did not affect selected tests of cognitive function, brain activations during cognitive control and functional brain connectivity were impaired on fMRI. The long-term clinical implications of these changes are not known and warrant future study.

## Background

Prostate cancer is the most common non-skin cancer in American men, and almost half of patients with this disease receive androgen deprivation therapy (ADT) over the course of their disease [1,2]. Evidence suggests that ADT can cause fatigue, decreased sexual function, gynecomastia, osteoporosis, and metabolic changes [3], but the adverse effects of ADT on cognition remain unclear. Observational and randomized studies have examined a possible association between androgens and cognitive function, including a potentially protective effect of testosterone against age-related cognitive decline [3-8]. A review [9] of prior studies using traditional neuropsychological testing in patients with prostate cancer treated with ADT showed equivocal findings; results were interpreted as showing either no effect [10], impaired function [11-13], or a mixed effect of ADT (with patients improving on some tests but doing worse on others) [14].

Functional magnetic resonance imaging (fMRI) provides a non-invasive method to assess brain activations during cognitive performance as well as the integrity of regional functional connectivities at rest. Clinicians have used fMRI to understand whether specific brain processes are impaired in patients with a medical (e.g., congenital heart disease [15]) or neurological condition (e.g., cerebral palsy [16]). Studies using fMRI have demonstrated etiology-specific changes in regional brain activations in patients with neurological or psychiatric conditions, even when they did not score differently from control individuals in neurocognitive tasks [17-19]. Thus, fMRI provides a more sensitive measure of brain functioning than neurocognitive testing alone.

Investigators have used fMRI and other imaging methods to evaluate the effects of chemotherapy and hormonal therapy on brain function in women with breast cancer [20]. For example, altered cortical and subcortical metabolism was found in breast cancer survivors 5-10 years after receiving adjuvant chemotherapy [21], and a patient who was diagnosed with breast cancer and underwent chemotherapy showed more white matter hyperintensities and altered spatial extents of brain activation than her non-affected, monozygotic twin, despite only small differences in working memory performance [22]. In addition, women with breast cancer taking tamoxifen showed widespread cortical hypometabolism on PET and MRI, when compared with scans in women not taking tamoxifen [23]. A longitudinal study in pre-menopausal women with early-stage breast cancer demonstrated changes in cognitive functioning and cerebral white matter integrity after chemotherapy [24]. Another recent study demonstrated significantly reduced activation in the left middle dorsolateral prefrontal cortex and premotor cortex in women with breast cancer compared with healthy controls irrespective of treatment history [25].

Our objective was to evaluate the impact of ADT on cognition using fMRI to study brain activations during a cognitive task and at rest, the stop-signal-task to study cognitive control, and the N-back test to study working memory. As a secondary outcome, we also evaluated quality of life.

## Methods

### Participants and baseline clinical assessments

First, patients with non-metastatic prostate cancer at the VA Connecticut Healthcare System from 01/2009 through 12/2010 were identified. All men who were prescribed ADT—either as adjuvant treatment or because of biochemical recurrence—were approached for participation. As per clinical practice, ADT consisted of medical castration with Goserelin 10.8 mg subcutaneously every 90 days for 6 months, after a lead-in period with Bicalutamide 50 mg daily. Patients with non-metastatic prostate cancer who had never been treated with ADT were evaluated as potential controls, with matching based on age and level of education. Exclusion criteria were: active second malignancy; Eastern Cooperative Oncology Group Performance Status >1; any significant cardiovascular conditions (e.g., unstable angina, pacemaker); hepatic, renal, or neurological disease; any investigational agents; or a score of less than 27 out of 30 on the mini-mental state examination (MMSE) [26]. Patients who had a history of axis-I psychiatric or substance (excluding nicotine) use disorders [27] were also excluded. All participants underwent a health questionnaire interview to ensure eligibility for fMRI. Participants who had had a prostatectomy were at least 3 months from their surgery and had fully recovered before study entry. Participants who were to receive radiation to the prostate underwent baseline assessment before starting any treatment and were also evaluated 3 to 4 months after completion of radiation treatment.

Our study was approved by the Yale Human Investigation Committee and the Human Investigation Subcommittee of the Veterans Affairs (VA) Connecticut Health Care System.

### Assessments

The ADT-treated participants were studied prior to initiation of ADT and again after 6 months of ADT, with effective castration documented by measuring testosterone level. Each individual assessment (questionnaire, performance-based, or radiological), as well as the combination of assessments (consisting of cognitive testing during a fMRI scan), are described below.

#### Quality of life

As a general assessment of overall status, participants completed standardized Quality-of-Life-Questionnaires (QOL) for prostate cancer patients (Fact-P^©^) at baseline and again at 6 months [28].

#### N-back (working memory) task

Working memory is a form of short-term memory that allows individuals to retain and manipulate information concurrent with engaging in complex tasks such as comprehension, reasoning, and learning. A behavioral paradigm widely used to study working memory is the N-back task [29,30]. In response to a series of letters displayed at a rate of 1 every 2 seconds, a higher accuracy rate and shorter reaction (RT) of correct trials represents better working memory. Our participants performed an N-back working memory task outside the MRI scanner at baseline and also at 6 months.

#### Stop signal (cognitive control) task

The stop signal task has been validated as a method to study cognitive control [31,32]. The stop signal task has two different stimuli randomly mixed in presentation and time intervals: a “Go” stimulus that instructs participants to respond by pressing a button, and a “Stop” stimulus that instructs participants to withhold their response. Pressing a button prematurely, prior to the appearance of the “Go” stimulus, terminates a trial. Likewise, a trial terminates at button press or when 1 second has elapsed after the appearance of the stop signal. A greater accuracy rate of “Go” and “Stop” trials indicates better cognitive control. Furthermore, in the stop signal task, participants can slow down in their Go trial response after they encounter a Stop error. This phenomenon, termed “post-error slowing”, is a useful index of how well participants are monitoring their own performance. Both inhibitory control and performance monitoring are critical to cognitive control. Our participants performed the SST during fMRI, both at baseline and at 6 months.

### Imaging protocol

Participants underwent fMRI during a resting state and while performing the stop signal task (as noted above). Functional blood oxygen level dependent (BOLD) signals were acquired using a 3 T scanner. Details of the imaging protocol and statistical modeling of brain images during stop signal task and at rest were as described previously [33]. Briefly, functional regions of interest were defined based on activated clusters from whole brain analysis. One of the main activated clusters was the medial prefrontal cortex (MPFC) which, as demonstrated in earlier work, plays a critical role in cognitive control [34,35].

### Statistical analysis

We estimated the sample size required to observe a group-by-time interaction effect on the basis of a previous pharmacological fMRI study [36]. The MPFC showed an effect size of 0.414 for the identical contrast in the stop signal task, according to Cohen’s f-hat = square root of [(Df/N)*(F-1)] [37]. The standard deviations for the interaction and within-cell error were 0.83 and 1.12, respectively. Using these results, and for a Type I error rate = 0.05 as well as assuming a balanced design, a sample size of 16 in each cell will have a power of 85% to detect group by time interaction. In a region of interest analysis, we derived the effect size (extent) of MPFC activation of stop > go trials [38]. Analyses were done based on the signals acquired; thus, “blinding” of results according to treatment group was not relevant. A significant effect of ADT would manifest as differences in regional brain activations for the contrast “(follow-up minus baseline in controls) > (follow-up minus baseline among patients receiving ADT)”. Signals obtained by fMRI from a task during rest can provide valuable information about the integrity of brain function, reflecting the intrinsic functional organization of the brain [39,40]. We analyzed the resting state BOLD signals with the MPFC as the region of interest.

For further details on N-back working memory task, stop signal task, imaging protocol and imaging data analysis during stop signal task and resting state please see Additional file 1.

## Results

From 01/2009 to 1/2011, 18 patients with non-metastatic prostate cancer receiving ADT and 18 control patients not receiving ADT were enrolled. One man died of a cause unrelated to prostate cancer and five withdrew their participation, resulting in 15 study participants undergoing ADT and 15 control participants (Table 1). These 30 study participants completed all scheduled assessments.

**Table 1:** Patient characteristics and Quality of life rating

**Group**	**ADT n = 15**	**Control n = 15**
Age	69.0 ± 5.3 y	66.1 ± 6.2 y
Education	9^th^ grade: 1	9^th^ grade: 1
	High School/GED: 5	High School/GED: 5
	College 1-3 years: 3	College 1-3 years: 3
	College graduate: 2	College graduate: 3
	Post-graduate: 4	Post-graduate: 3
MMSE	29.0 ± 1.3	29.5 ± 0.6
Cancer staging	Stage II: 13	Stage I: 1
	Stage III: 2	Stage II: 12
		Stage III: 2
Local therapy	Radiation 100%	Radiation 26.7%
		Surgery 66.7%
		Surgery + Radiation 6.7%
QOL at baseline	115 ± 22	132 ± 18
QOL of life at 6 months	110 ± 23	128 ± 21
Testosterone at 6 months	0.14 ± 0.10 ng/ml	2.84 ± 0.94 ng/ml

Note: ADT: Androgen Deprivation Therapy; GED: General Education Development Test; MMSE: Mini Mental State Examination; QOL: FACT-P quality of life score; there is no difference between groups in age (t = 1.172, p = 0.588, two-sided two-sample t test); there is no difference between groups in MMSE score (t = 1.417, p = 0.167, two-sided two-sample t test); staging follows the current guidelines of the 2010 American Joint Committee on Cancer (AJCC) that include Gleason score in the staging; there is also no difference across the two time points in the change of QOL between groups (F = 0.056, p = 0.814, interaction, repeated measures analysis of variance).

### Quality of life scores

No statistically significant differences between ADT patients and control patients were found in QOL scores at baseline, or with regard to change over 6 months using the FACT-P^©^ questionnaire (Table 1). Of note, FACT-P^©^ does not specifically assess cognitive function.

### Cognitive performance in N-back task and stop signal task

As measured by the N-back task or by the stop-signal-task, no statistically significant differences in cognitive performance were observed at 6 months compared to baseline in either group (Tables 2 and 3).

**Table 2:** Performance in the N-back working memory task

	**0-back (correct %)**	**0-back (RT, ms)**	**1-back (correct %)**	**1-back (RT, ms)**	**2-back (correct %)**	**2-back (RT, ms)**
ADT_B	98 ± 3	545 ± 114	85 ± 13	668 ± 147	63 ± 15	746 ± 159
ADT_F	97 ± 5	571 ± 91	86 ± 11	704 ± 165	64 ± 17	787 ± 173
Control_B	99 ± 2	491 ± 88	85 ± 14	585 ± 157	75 ± 17	677 ± 152
Control_F	99 ± 1	540 ± 87	89 ± 3	626 ± 139	78 ± 13	714 ± 126
P value*	0.402	0.389	0.577	0.932	0.493	0.920

Note: B: baseline; F: follow-up; RT = reaction time of correct trials; *P value of the group by time interaction in repeated measures analysis of variance.

**Table 3:** Behavioral performance in the stop signal task

	**SSRT (ms)**	**Median go RT (ms)**	**%go**	**%stop**	**PES (effect size)**
ADT_B	267 ± 71	697 ± 127	90.9 ± 5.5	56.5 ± 6.0	0.83 ± 1.72
ADT_F	278 ± 37	678 ± 123	89.6 ± 5.2	53.1 ± 3.7	1.21 ± 1.12
Control_B	260 ± 57	676 ± 122	91.6 ± 5.9	56.5 ± 4.5	1.73 ± 1.56
Control_F	268 ± 56	705 ± 115	91.2 ± 5.4	56.9 ± 5.8	1.49 ± 1.35
P value*	0.480	0.243	0.567	0.051	0.310

Note: B: baseline; F: follow-up; SSRT: stop-signal reaction time; go RT: reaction time of correct go trials; %go and %stop: percentage of successful go and stop trials; PES: post-error slowing; all values are mean ± standard deviation; *P value of the group by time interaction in repeated measures analysis of variance

Table 2 shows the results of N-back task performance scores. Overall, correct response rate decreased with increasing memory load for both control and ADT participants. Three-way repeated measures ANOVA with time (baseline vs. follow-up) and load (0-, 1-, and 2- back) as within-subject variables, and group (control vs. ADT) as the between-subject variable, showed that the interaction was not significant for hit rate (p = 0.989) or reaction time (RT) (p = 0.948) within the correct trials. Three-way repeated measures ANOVA with time (baseline vs. follow-up) and load (0- vs. 1- back, or 0- vs. 2-back) as within-subject variables, and group (control vs. ADT) as the between-subject variable, also showed that none of the interactions were significant (p = 0.859 and p = 0.934, respectively, for 0- vs. 1- back and 0- vs. 2- back re: hit rate; p = 0.814 and p = 0.732, respectively, for 0- vs. 1- back and 0- vs. 2- back re: RT of correct trials). We conducted two-way repeated measures ANOVA separately for 0-, 1-, and 2- back data, which again yielded non-significant interactions (Table 2). These results indicated indistinguishable N-back task performance between the two groups across the two time points.

Table 3 shows the performance outcomes of the stop signal task for the ADT and control group, at baseline and at 6 months. We recorded the Go success rate, Stop success rate, reaction time (RT) of Go success trials, the stop signal reaction time (SSRT), as well as the effect size of post-error slowing (i.e., how much participants slowed down in a Go trial following a Stop error), as an index of performance monitoring. The results showed that there were no differences in performance at 6 months compared to baseline in either group.

### Brain activations during cognitive control using the stop-signal-task

**Figure 1:**
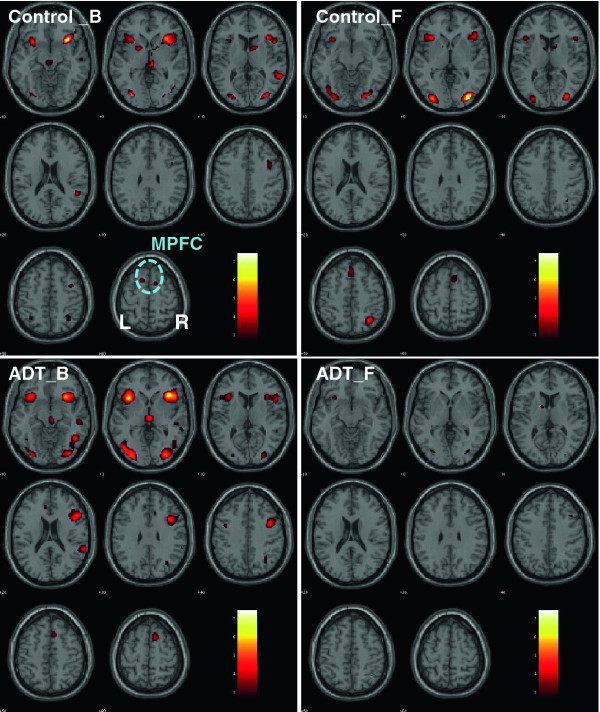
**Regional brainactivations duringcognitive controlwhile performingthe stopsignal task.** ADT = patients who received 6 months of androgen deprivation therapy; Controls = patients who did not receive any hormonal therapy; B = baseline; F = at 6- month follow-up. The significance of activation, as reflected by a map of T values (color bar), is shown here on a structural brain image in axial sections, from z = -10 to z = +60, with adjacent sections 10 mm apart. Neurological orientation: Right (R) = right. Note that ADT patients showed diminished activations in a number of brain regions at 6 month follow-up, including the medial prefrontal cortex (MPFC), an area critical for cognitive control.

**Figure 2:**
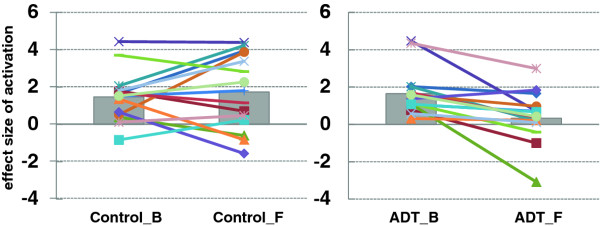
**The effectsize ofmedial prefrontalcortical (MPFC)activations.** Control patients are shown on the left panel; ADT patients are shown on the right panel; B = baseline; F = at 6-month follow-up. In each panel, each symbol and line represents the data of an individual patient. The gray bars indicate the mean value of the effect sizes of MPFC activations. Individuals varied in the change of MPFC activations from baseline to follow-up, but, on average, the ADT group showed significant decrease in MPFC activations after 6 months of ADT, as compared to the control group.

Brain activations while performing the stop-signal-task were similar between baseline and follow-up for the control group. In contrast, regional brain activations were significantly diminished in the ADT group at follow-up, as compared to baseline (Figure 1). These differences were most prominent in the medial prefrontal cortex (MPFC), right insula, and right middle/inferior frontal cortices. As previous work has demonstrated, the prefrontal cortex plays a critical role in cognitive control [34,35]. In a region of interest analysis, we focused on the MPFC and extracted the effect size of activation for individual participants. A repeated measures analysis of variance, with the ADT vs. control as the between-subject factor and follow-up vs. baseline as the within-subject factor, showed that ADT significantly decreased medial prefrontal cortical activation during cognitive control (F_1,28_ = 7.903, p = 0.009, group by time interaction, Figure 2).

### Functional connectivity of medial prefrontal cortex during a resting state

Despite performance scores on N-back task and stop-signal-task that were similar to the control patients, ADT patients showed decreased MPFC brain activations on fMRI at 6 months while performing the stop signal task. We were interested in whether changes on fMRI could also be observed when patients were not performing any cognitive tasks and studied the correlation of low frequency BOLD signals between the MPFC and other brain regions during a resting state. This resting state imaging reflects the functional connectivity between different brain regions and measures how well individual brain regions activate in a concerted manner as an important index of the integrity of brain functions.

**Figure 3:**
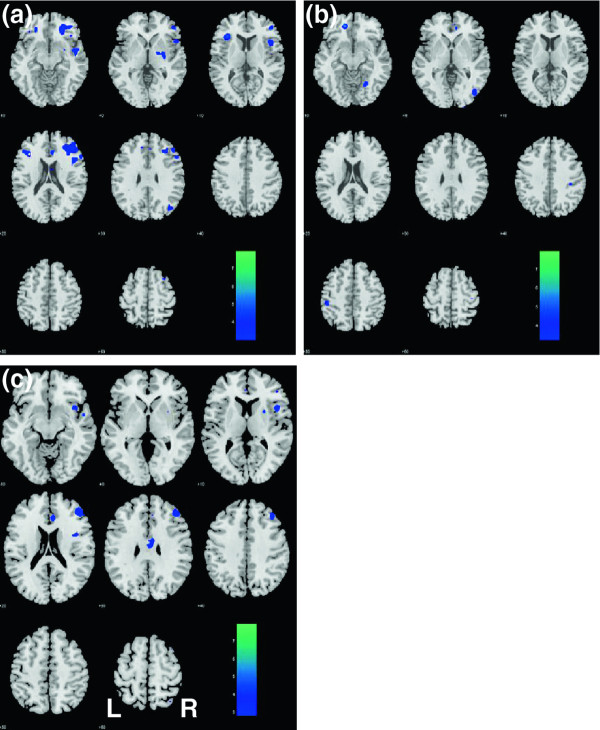
**Changes inresting statefunctional connectivitywith themedial prefrontalcortex asresult ofADT.** After 6 months of ADT, patients showed decreased connectivity with the dorsolateral prefrontal cortex (DLPFC), rostral anterior cingulate cortex, and the insula, all on the right side, as compared to control patients. **(a)** ADT_B > ADT_F; **(b)** Control_B > Control_F; **(c)** (Control_F > Control_B) – (ADT_F > ADT_B). B: baseline; F: follow-up. The significance of difference, as reflected by a map of T values (color bar), is shown here on a structural brain image in axial sections, from z = -10 to z = +60, with adjacent sections 10 mm apart. Neurological orientation: Right (R) = right.

The results of an analysis of variance showed that, compared with the control group, ADT patients had at 6 months a decrease in MPFC connectivity (p < 0.001, uncorrected; Figure 3) with the dorsolateral prefrontal cortex (DLFPC), right insula, right superior temporal gyrus, and the rostral anterior cingulate cortex, structures that are broadly implicated in cognitive control.

## Discussion

Studies of traditional neurocognitive testing without brain imaging have shown equivocal impact of ADT on cognitive function. In the current study, we found suppressive effects on brain activations and disruption in functional connecticity on fMRI in prostate cancer patients after 6 months of ADT. Interestingly, performance in neurocognitive testing and their QOL scores remained similar compared to their baseline, and control patients without ADT did not show any fMRI changes. Our patients were matched by age and level of education, and all assessments were performed either before or at least 3 months after any surgery or radiation treatment to minimize any impact of treatment-related symptoms.

The stability of cognitive performance scores after six months of ADT is consistent with a recent report [41] that used neurocognitive tesing alone in a larger sample of patients receiving twelve months of ADT. In contrast, the changes we found for brain activations on fMRI while performing the stop-signal-task after 6 months of ADT were substantial.

Previous work has demonstrated that the MPFC and a network of brain regions interacting with the MPFC play a critical role during cognitive control [34,35]. The findings of altered MPFC activation following ADT are consistent with previous literature on the modulatory effects of androgen on cerebral cortical activities [42-46]. For example, a higher level of free testosterone was associated with greater cerebral blood flow in the hippocampus and prefrontal cortices in elderly men [45], testosterone replacement therapy increased cerebral blood perfusion in the midbrain and prefrontal cortex in hypogonadal men [42], and administration of testosterone increased ventral striatal responses to reward in healthy women [44]. A preliminary report of decreased parieto-occipital brain activation during visuospatial processing in five men undergoing ADT [47] is consistent with our results in suggesting a suppressive effect of ADT on brain activity.

As mentioned previously, neuroimaging studies have reported differences in regional brain activations in neurological or psychiatric patients even when they performed at a level equal to their demographically-matched control participants. These *performance*-*independent* changes in brain activities cannot be accounted for by effort or motivation, and potentially represent the neural correlates specific to the cerebral pathologies. Similarly, the findings of decreased cortical activations in prostate cancer patients undergoing ADT, despite similar performance scores on neurocognitive testing, indicate that changes in brain activations were not a result of these patients being less engaged in the task, as compared with their counterparts. Furthermore, neurocognitive performance is potentially subject to practice effects whereby participants’ performance improves upon repeated testing. Performance-independent differences in brain activations may thus be more objective and reveal subtle changes in brain function that cannot be measured by neurocognitive testing alone.

Limitations of this study include a small sample size and a limited duration of ADT exposure, yet we were able to demonstrate an impact of therapy. Although our study design supports the validity of our results, the results should be considered preliminary with the need for replication in future work. Our study is distinctive in evaluating the effects of ADT using functional imaging; it also is novel in demonstrating a disruption of functional connectivity at rest in patients treated with ADT. In addition, although our study was not a randomized trial, the potential impact of ADT on brain activation should be detectable using either randomized or observational approaches. Finally, our study cannot answer questions such as whether the differences observed using fMRI would worsen over time with or without continued ADT, or whether even these current effects would eventually lead to clinically apparent signs or symptoms. Future studies with longer follow-up are warranted to answer these important questions.

## Conclusion

ADT limited to 6 months did not affect scores on selected tests of cognitive function. However, using sensitive techniques involving fMRI, ADT for 6 months suppressed brain activations during cognitive control and disrupted brain functional connectivity in prostate cancer patients. The long-term clinical implications of these changes are not known and warrant future study.

## Competing interests

The authors declare that they have no competing interests.

## Authors’ contributions

HHC conceived of the study, participated in its design and coordination and drafted the manuscript. EU participated in the design and enrollment of patients and helped to draft the manuscript. SZ, SH and SRB participated in the coordination of the study and performed the statistical analysis of neurocognitive testing. XL performed the analysis of imaging data. MR and JC participated in the design of the study and the writing of the manuscript. CRL participated in the design of the study, data analysis and writing of the manuscript. All authors read and approved the final manuscript.

## Pre-publication history

The pre-publication history for this paper can be accessed here:

http://www.biomedcentral.com/1471-2407/12/371/prepub

## Supplementary Material

Additional file 1 Supplemental material. Click here for file

## Author details

^1^Department of Internal Medicine & Yale Comprehensive Cancer Center, Yale University School of Medicine, New Haven, CT 06519, USA. ^2^Medical Service, VA Connecticut Health Care System, West Haven, CT 06516, USA. ^3^Tower Research, Los Angeles, CA 90048, USA. ^4^Department of Psychiatry, Yale University School of Medicine, New Haven, CT 06519, USA. ^5^Department of Biostatistics, Brown University School of Medicine, Providence, RI 02912, USA.^6^Clinical Epidemiology Research Center, West Haven, CT 06516, USA.^7^Department of Neurobiology, Yale University School of Medicine, New Haven, CT 06520, USA. ^8^Interdepartment Neuroscience Program, Yale University, New Haven, CT 06520, USA. ^9^Cancer Center, VA Connecticut Health Care System, 950 Campbell Avenue, West Haven, CT 06516, USA.
