# Supplementary material for: Effects of androgen deprivation on brain function in prostate cancer patients – a prospective observational cohort analysis
Source: BMC Cancer. 2012 Aug 27;12:371. doi: 10.1186/1471-2407-12-371 (PMC3502584; doi:10.1186/1471-2407-12-371)
Supplement: Additional file 1 — Supplemental material. [file 1471-2407-12-371-S1.doc]

**Supplemental Material**

**Effects of Androgen Deprivation on Brain Function in Prostate Cancer Patients – A Prospective Observational Cohort Analysis**

Herta H. Chao,^1,2,*^  Edward Uchio,^3^ Sheng Zhang,^4^ Sien Hu,^4^ Sarah R. Bednarski,^4^ Xi Luo,^5^ Michal Rose, ^1,2^, John Concato, ^1,2,6^ Chiang-shan R. Li,^4,7,8^

^1^Dept. of Internal Medicine & Yale Comprehensive Cancer Center, Yale University School of Medicine, New Haven, CT 06519, USA

^2^Medical Service, VA Connecticut Health Care System, West Haven, CT 06516, USA

^3^Tower Research, Los Angeles, CA 90048

^4^Dept. of Psychiatry, Yale University School of Medicine, New Haven, CT 06519, USA

^5^ Dept. of Biostatistics, Brown University School of Medicine, Providence, RI 02912, USA

^6^Clinical Epidemiology Research Center, West Haven, CT 06516, USA

^7^Dept. of Neurobiology, Yale University School of Medicine, New Haven, CT 06520, USA

^8^Interdepartment Neuroscience Program, Yale University, New Haven, CT 06520, USA

**METHODS:**

**N-back working memory task [e1,e2]:**

The N-back task required participants to respond to a series of letters presented at a rate of 1 every 2 s (stimulus duration = 500 ms). Fifteen phonologically distinct letters served as stimuli (A, B, C, D, E, F, G, H, K, M, N, P, S, W, X), with three blocks in the task, differing in working memory load. In the 1- and 2-back blocks, the participants responded by pressing a button when the current letter matched the letter from one and two steps earlier, respectively, in the sequence (e.g., B as target letter in sequence A-B-B for 1-back, and B-A-B for 2-back). In a 0-back block, participants pressed a button whenever they saw letter “X”.

Each subject performed 3 sessions of the N-back task, with each session comprising two each of 0-, 1-, and 2- back blocks, the order of which was counter-balanced across sessions (Session 1: 0-, 1-, 2-, 1-, 2-, 0- back; Session 2: 1-, 0-, 2-, 2-, 1-, 0- back; Session 3: 2-, 0-, 1-, 0, 2-, 1- back). Each block began with an information screen showing the “load” for that block (5 s) and contained 24 trials, with one-third of them representing targets. Correct response rate and reaction time of correct trials were recorded for each block and averaged for the each load condition for analyses.

**Stop-signal-task [e3,e4]:**

We used the stop-signal-task (SST) to examine possible ADT-induced changes in regional brain activations during cognitive control. Two trial types were used: “Go” and “Stop,” randomly mixed in presentation. A small dot appeared on the screen to engage attention at the beginning of a Go trial. After a randomized time interval (fore-period) anywhere between 1 and 5 s, the dot turned into a circle, prompting the participants to quickly press a button. The circle vanished at button press or after 1 second elapsed, whichever came first, and the trial terminated. A premature button press prior to the appearance of the circle also terminated the trial. Approximately three quarters of all trials were Go trials and the remainder were Stop trials. In a Stop trial, an additional “X,” the “stop” signal, appeared after the go signal. The participants were instructed to withhold button press upon seeing the stop signal. Likewise, a trial terminated at button press or when 1 second elapsed since the appearance of the stop signal. The time interval between the stop and the go signals (or the stop-signal delay, SSD) started at 200 milliseconds and varied from one Stop trial to the next according to a staircase procedure, increasing and decreasing each by 67 milliseconds with a successful and failed stop, respectively. There was an inter-trial interval of 2 s. Participants were instructed to respond to the go signal quickly while keeping in mind that a stop signal could come up in a small number of trials. Prior to fMRI each subject had a practice session outside the scanner. Each subject completed three 8-minute runs of the task.

With the staircase procedure, a “critical” SSD was computed that represented the time delay required for the subject to succeed in withholding a response in the Stop trials half of the time [5]. We then estimated the stop signal reaction time (SSRT) for each individual subject/fMRI session by subtracting the critical SSD from the median Go trial reaction time (RT)[3,4]. The SSRT described the time one needs to inhibit a prepotent response; short SSRT indicated better capacity of inhibitory control. In a reaction time task, participants typically slowed down in response after they committed an error [e6,e7]. By comparing the RT of Go trials following a stop error and those following another Go trial, we obtained a measure of post-error slowing as an index of performance monitoring.

**Sample size calculation:**

We estimated the sample size required to observe a group-by-time interaction effect on the basis of a previous pharmacological fMRI study [e8]. The medial prefrontal cortex (MPFC) showed an effect size of 0.414 for the identical contrast in the stop signal task, according to Cohen’s f-hat = square root of [(Df/N)*(F-1)] [e9]. The standard deviations for the interaction and within-cell error were 0.83 and 1.12, respectively. Based on these results,and for a Type I error rate=0.05 as well as assuming a balanced design, a sample size of 16 in each cell will have a power of 85% to detect group by time interaction. In a region of interest analysis, we derived the effect size (extent) of MPFC activation of stop > go trials [e10]. A significant effect of ADT would manifest as differences in regional brain activations for the contrast “(follow-up minus baseline in controls) > (follow-up minus baseline among patients receiving ADT)”.

**Imaging protocol:**

Conventional T_1_-weighted spin echo sagittal anatomical images were acquired for slice localization using a 3T scanner (Siemens Trio). Anatomical images of the functional slice locations were obtained with spin echo imaging in the axial plane parallel to the Anterior Commissure-Posterior Commisure (AC-PC) line with Repetition Time (TR) = 300 ms, Echo Time (TE) = 2.5 ms, bandwidth = 300 Hz/pixel, flip angle = 60 degrees, field of view = 220x220 mm, matrix = 256x256, 32 slices with slice thickness = 4mm and no gap. Functional, blood oxygen level dependent (BOLD) signals were then acquired with a single-shot gradient echo echoplanar imaging (EPI) sequence. Thirty-two axial slices parallel to the AC-PC line covering the whole brain were acquired with TR = 2,000 ms, TE = 25 ms, bandwidth = 2004 Hz/pixel, flip angle = 85 degrees, field of view = 220x220 mm, matrix = 64x64, 32 slices with slice thickness = 4mm and no gap. Two-hundred-and-forty images were acquired in each run for a total of 3 runs.

In another 8-minute run of the fMRI scan, we collected BOLD data with the participants eyes closed and resting in the scanner. They were instructed to relax and not think about the experiment or anything stressful, in accord with established routines of “resting state” scans [e11]. The resting state scans provided data for connectivity analyses, to evaluate whether and how brain regions are integrated in functioning when individuals are not engaged in a behavioral task.

**Imaging data analysis: stop-signal-task**

Data were analyzed with Statistical Parametric Mapping 8 (Wellcome Department of Imaging Neuroscience, University College London, U.K.). Images from the first five TRs at the beginning of each trial were discarded to enable the signal to achieve steady-state equilibrium between RF pulsing and relaxation.

Details of image pre-processing and statistical analyses were as described in our previous work [e4]. Briefly, images of each individual subject were first corrected for slice timing and realigned (motion-corrected). A mean functional image volume was constructed for each subject for each run from the realigned image volumes. These mean images were normalized to an MNI (Montreal Neurological Institute) EPI template, with affine registration followed by nonlinear transformation [e12,e13]. The normalization parameters determined for the mean functional volume were then applied to the corresponding functional image volumes for each subject. Finally, images were smoothed with a Gaussian kernel of 10 mm at Full Width at Half Maximum. The data were high-pass filtered (1/128 Hz cutoff) to remove low-frequency signal drifts.

A statistical analytical design was constructed for each individual subject, using the general linear model (GLM) with the onsets of go signal in each of go and stop trials convolved with a canonical hemodynamic response function (HRF) and with the temporal derivative of the canonical HRF and entered as regressors in the model [e14]. Realignment parameters in all 6 dimensions were also entered in the model. Serial autocorrelation caused by aliased cardiovascular and respiratory effects was corrected by a first-degree autoregressive or AR(1) model. The GLM estimated the component of variance that could be explained by each of the regressors. In the first-level analysis, we constructed for each individual subject one statistical contrast: stop > go. The con or contrast (difference in β) images of the first-level analysis was then used for the second-level group statistics (random effect analysis or RFX, [e15]).

Activated brain regions were identified using an atlas [e16]. Functional regions of interest were defined based on activated clusters from whole brain analysis. We derived the effect size (extent) of media prefrontal cortical activation of stop > go trials for individual participants [e17]. In a repeated measures analysis of variance (ANOVA) of the effect size with group (ADT vs. control) as a between-subject variable and fMRI session (baseline vs. follow-up) as a within-subject variable, we examined the group by session interaction effect. Specifically, a significant effect of ADT would manifest as differences in regional brain activations for the contrast “(follow-up minus baseline in controls) > (follow-up minus baseline among patients receiving ADT)”.

**Imaging data analysis: resting state**

Recent advances in brain imaging suggest that the fMRI signals obtained during rest can provide valuable information about the integrity of brain functions. That is, while a behavioral task evokes transient regional brain activations that are captured by high frequency signal changes, the low frequency signals obtained when subjects are resting reflect the intrinsic functional organization of the brain [e18,e19]. Specifically, low frequency blood oxygenation level dependent (BOLD) signal fluctuations reflect connectivity between functionally related brain regions. Studies of this “spontaneous” activity have provided insight into the intrinsic functional architecture of the brain and show that the spontaneous fluctuations are present in many neuroanatomical systems, including motor, visual, auditory, default mode, memory, language, dorsal attention, and ventral attention systems [e18,e19].

Resting state BOLD signals underwent the same pre-processing as did the task-related data. Additional preprocessing was applied to reduce spurious BOLD variances that were unlikely to reflect neuronal activity [e18,e19]. The sources of spurious variance were removed through linear regression, by including the signal from the ventricular system, the white matter, and the whole brain, in addition to the six parameters obtained by rigid body head motion correction. First-order derivatives of the whole brain, ventricular, and white matter signals were also included in the regression. Cordes and colleagues suggested that BOLD fluctuations below a frequency of 0.1Hz contribute to regionally specific BOLD correlations [e20]. The majority of resting state studies low-pass filtered BOLD signal at a cut-off of 0.08 or 0.1 Hz [e19]. Thus, we applied a temporal band-pass filter (0.009Hz < f < 0.08Hz) to the time course in order to obtain low-frequency fluctuations.

To elucidate potential changes in functional connectivity as a result of ADT, we focused on a region of the medial prefrontal cortex which, as demonstrated in our earlier work, played a critical role in cognitive control [e21,e22]. The BOLD time courses were averaged spatially over this region of interest for each subject. For individual participants, we computed the correlation coefficient between the averaged time course of each seed region and the time courses of all other brain voxels. To assess and compare the resting state “correlograms,” we converted these image maps, which were not normally distributed, to z score maps by Fisher’s z transform [23]: $z=0.5\log_{e}[(1+r)/(1-r)]$. The Z maps were used in group random effect analyses for an interaction effect of “(follow-up minus baseline in controls) > (follow-up minus baseline among patients receiving ADT)”.

**REFERENCES**

[e1] Baddeley A. (2003) Working memory: looking back and looking forward. Nature Reviews. Neuroscience 4: 829–839.

[e2] Kirchner WK. (1958) Age differences in short-term retention of rapidly changing information. Journal of Experimental Psychology, 55: 352-358.

[e3] Logan GD, Cowan WB, Davis KA. (1984) On the ability to inhibit simple and choice reaction time responses: a model and a method. J Exp Psychol Hum Percept Perform. 10:276-291.

[e4] Li C-SR, Huang C, Constable T, Sinha R (2006) Imaging response inhibition in a stop signal task – neural correlates independent of signal monitoring and post-response processing. J Neurosci 26: 186-192.

[e5] Levitt H (1970) Transformed up-down methods in psychoacoustics. J Acoust Soc Am 49: 467-477.

[e6] Rabbit PMA (1966) Errors and error correction in choice-response tasks. J Exp Psychol 71: 264-272.

[e7] Li CS, Huang C, Yan P, Paliwal P, Constable RT, Sinha R. (2008) Neural correlates of post-error slowing during a stop signal task: a functional magnetic resonance imaging study. J Cogn Neurosci. 20: 1021-1029.

[e8] Li CS, Morgan PT, Matuskey D, Abdelghany O, Luo X, Chang JL, Rounsaville BJ, Ding YS, Malison RT (2010) Biological markers of the effects of intravenous methylphenidate on improving inhibitory control in cocaine-dependent patients. Proc Natl Acad Sci U S A. 107:14455-14459.

[e9]Cohen J (1988). Statistical Power Analysis for the Behavioral Sciences (second edition). Lawrence Erlbaum Associates.

[e10] Brett M, Anton J-L, Valabregue R, Poline J-P. 2002. Region of interest analysis using an SPM toolbox. Abstract presented at the 8^th^ International Conference on Functional Mapping of the Human Brain. June 2--6; Sendai, Japan. Available on CD-ROM in Neuro-Image, 16(2), abstract 497.

[e11] Raichle ME, MacLeod AM, Snyder AZ, Powers WJ, Gusnard DA, Shulman GL. (2001) A default mode of brain function. Proc Natl Acad Sci U S A 98: 676-682.

[e12] Ashburner J, Friston KJ. (1999) Nonlinear spatial normalization using basis functions. Hum Brain Mapp 7:254-266.

[e13] Friston KJ, Ashburner J and Frith CD, Polone J-B, Heather JD, Frackowiak RSJ (1995a) Spatial registration and normalization of images. Hum Brain Mapp 2:165-189.

[e14] Friston KJ, Holmes AP, Worsley KJ, Poline J-B, Frith CD, Frackowiak RSJ (1995b) Statistical parametric maps in functional imaging: a general linear approach. Hum Brain Mapp 2:189-210.

[e15] Penny WD, Holmes, A.P.,Friston, K.J. (2004) Random-effects analysis. In: Human Brain Function, 2nd Edition (R.S.J. Frackowiak KJF, C. Frith, R. Dolan, K.J. Friston, C.J. Price, S. Zeki, J. Ashburner,W.D.Penny, ed), pp 843-850: Academic Press.

[e16] Duvernoy HM. 2003. The Human Brain, Second edition. Wien (Austria): Springer-Verlag

[e17] Brett M, Anton J-L, Valabregue R, Poline J-P. 2002. Region of interest analysis using an SPM toolbox. Abstract presented at the 8^th^ International Conference on Functional Mapping of the Human Brain. June 2--6; Sendai, Japan. Available le on CD-ROM in Neuro-Image, 16(2), abstract 497.

[e18] Fair DA, Schlaggar BL, Cohen AL, Miezin FM, Dosenbach NU, Wenger KK, Fox MD, Snyder AZ, Raichle ME, Petersen SE (2007) A method for using blocked and event-related fMRI data to study "resting state" functional connectivity. Neuroimage 35: 396-405.

[e19] Fox MD, Raichle ME. (2007) Spontaneous fluctuations in brain activity observed with functional magnetic resonance imaging. Nat Rev Neurosci 8: 700-711.

[e20] Cordes D, Haughton VM, Arfanakis K, Carew JD, Turski PA, Moritz CH, Quigley MA, Meyerand ME (2001) Frequencies contributing to functional connectivity in the cerebral cortex in "resting-state" data. AJNR Am J Neuroradiol 22: 1326-1333.

[e21] Ridderinkhof KR, van den Wildenberg WP, Segalowitz SJ, Carter CS (2004) Neurocognitive mechanisms of cognitive control: the role of prefrontal cortex in action selection, response inhibition, performance monitoring, and reward-based learning. Brain Cogn. 56:129-140.

[e22] Rushworth MF. (2008) Intention, choice, and the medial frontal cortex. Ann N Y Acad Sci. 1124: 181-207.

[e23] Jenkins GM, Watts DG (1968) Spectral Analysis and Its Applications. Holden-Day, San Francisco.
